# Correspondence

**Published:** 1867-02

**Authors:** 


### Correspondence.

FORT EDWARD, January 28th, 1867.

*To the Editor of the Buffalo and Medical Surgical Journal:*

DEAR SIR:—I communicate the following cases of purpura-hæmorrhagicæ and hæmatemesis, successfully treated with large and repeated doses of tupentine, "the commercial spts." I am aware that turpentine has been employed by some as a hemastetic, but do not know that it has been particularly mentioned for its specific value in such cases. Some time since I received a hasty summons to visit, in consultation, a patient in an adjoining town. Upon arriving on the premises, I found the house filled with anxious spectators; the patient, an Irishman, aged 40 years, reported in a dying condition. The priests were about to administer the holy wafer. The attending physicians had abandoned the case as hopeless, and were not satisfied as to the nature of the disease.

After clearing the room of these dangerous, though well meaning sympathizers, I found patient in following condition: countenance pale and anxions; recognized me, but could not speak; pulse 140, feeble and irregular; a bloody mucus was oozing from the mouth, also blood from the nose, ears and eyelids; the entire mucous membrane of mouth was streaked with blood; gums soft and bleeding. Upon close observation, I could detect several rose-colored spots or petechiæ upon forehead and face. On turning down the bed-clothes, the body and legs presented a varigated appearance, petechiæ, interspersed with vibices-ecchymosis. Many of the spots were black and purple, often coalescing, and resembling skin burned with powder. I did not hesitate to pronounce this a pure case of the purpura-hæmorrhagicæ of Willan.

The patient had occasional vomiting of blood, accompanied with hiccough. As life seemed fast passing away, I lost no time in executing the following plan of treatment: I administered two ounces turpentine, with same quantity of caster oil. I repeated the dose in one-half hour, alternating with a little old wine. At the expiration of one hour, the vomiting and hiccough had ceased; the pulse began to revive. I now left him, with orders to continue the oil and turpentine, in one ounce doses each, for three consecutive hours, or until the bowels should have moved freely, giving a Dover's powder at night, and occasionally a little wine or sling.

I visited this patient at six o'clock following morning, twelve hours from the first attack. His bowels had been freely evacuated; his countenance wore a more intelligent appearance or expression; there was no more hemorrhage, except a slight quantity from expectoration and cough, and accompanied with some darting pains through the chest; he expressed himself as feeling much more comfortable; the rose spots on the body and limbs began to fade. Upon my third visit, the patient was so far improved that I left him to complete treatment with tinct. iron, gentian and bark, with Dover's powders to control all local irritation. He recovered rapidly, and in one week was able to resume business.

I have had, more recently, an opportunity to further prove the efficacy of large doses of turpentine in a case of hæmatemesis, *(vomitus-cruentus,)* or excessive hemorrhage of the stomach. The patient, aged 35 years, of a strumous diathesis, was seized with vomiting blood on stepping from his carriage to house. I saw him during the second attack of vomiting, which occurred at intervals of about five minutes. The blood first rejected had a dark and somewhat grumous appearance.

The quantity already thrown off seemed enormous and frightful. The patient was a man of industrious and strictly sober habits; had no warning or premonitions, except slight oppression at the stomach; had no pain, nor were there any indications of a gastritis; his pulse ran low and feeble; countenance pale. Viewing the case as an accidental circumstance, arising from the rupture of blood-vessels, or some mechanical obstruction of the liver, spleen, or other viscera, I immediately gave him from two to three ounces turpentine, with castor oil. He retained this some five minutes, when it came up, already filling a large pail, half full, besides that lost before reaching the house. I repeated the dose, and this he kept down ten or fifteen minutes, and when rejected, looked black and coagulated. I again repeated the dose for the third time, and this he did not throw off under half an hour. The quantity was much less, black, and resembled portions of liver and coffee grounds; appeared more comfortable and inclined to sleep. I ordered a full portion of oil, with one-half ounce turpentine, to be given in a little wine. From this time the vomiting entirely ceased. In about six hours the bowels had moved freely, the contents of which proved mostly coagulated blood. He remained feeble for a few days, but in a short time recovered, under a generous tonic treatment—iron, and the vegetable tonics, gentian and bark. I have found turpentine to be highly beneficial in all hemorrhages when administered in full and oft-repeated doses. Experience will yet prove this to be one of the most valuable remedial agents we now possess.

Most respectfully,

J. F. Norton, M. D.

---

To the Editor of the Buffalo Medical and Surgical Journal:

Dear Sir:—Inasmuch as the removal of obstructions from the nasal passages of children by the usual mode, as described in our books, is many times attended with perplexing difficulty and chagrin to the surgeon, and intense anxiety to friends, I am induced to offer a few suggestions as to a mode of practice quite different, which I have pursued for over twenty-five years, obviating many objections to the usual plan. Therefore, if you think proper, please publish in your Journal, the following simple mode, which in no instance, of all the varied forms of substances that I have found in the nasal passages, has it required over five minutes, to give the desired relief. It entirely obviates the use of chloroform, as the efforts and struggles of the patient rather facilitate the operation than otherwise.

The instrument consists of six or eight inches of fine wire, bent in the form of a hair-pin; indeed I have several times used a common hair-pin with very good results; yet a finer unannealed wire, is better. The doubled end of the wire should be larger or smaller, to correspond to the size of the nostril of the patient. The patient, (usually a small child) seated in a male assistant's lap, with his extremities secured, the surgeon can with a good degree of confidence thrust the bent end of the wire into the nostril above the obstruction, and withdraw it, or partially withdraw it, and in most instances the patient is immediately relieved. If not by the first effort, try again, as the plan is sure to succeed; at least it always has in my hands.

By the usual operation, before the age of chloroform, while in my pupilage, I saw this trifling accident several times by respectable surgeons converted into a protracted and tedious operation, which in all probability might have been done in a few moments with the bent wire. The safety in the use of the instrument without chloroform, and the despatch attending it, renders it in the opinion of the writer the best possible mode for this purpose.

I have several times before the introduction of chloroform into practice, used the same instrument to remove foreign bodies from the internal ear, with good results. Yet I now usually administer chloroform and use forceps as a better mode.

I am of the opinion, the same principle applied to obstructions of the œsophagus might give satisfactory results. The wire should be larger and longer, and the double at the end should be made to correspond to the size of the orifice. I can see no good reason why this plan might not be resorted to in the absence of other instruments, with a reasonable prospect of success.

This last is only a suggestion, not the result of experiment.

If the above suggestions induce a trial, especially on the nasal organs, the writer will in some degree have accomplished his object; as it is in his opinion, quite likely to be followed by adoption.

Yours, truly, M. H. SHAW,

Buffalo, N. Y.

